# Is cardiac involvement prevalent in highly trained athletes after SARS-CoV-2 infection? A cardiac magnetic resonance study using sex-matched and age-matched controls

**DOI:** 10.1136/bjsports-2021-104576

**Published:** 2021-11-29

**Authors:** Liliána Szabó, Vencel Juhász, Zsófia Dohy, Csenge Fogarasi, Attila Kovács, Bálint Károly Lakatos, Orsolya Kiss, Nóra Sydó, Emese Csulak, Ferenc Imre Suhai, Kristóf Hirschberg, Dávid Becker, Béla Merkely, Hajnalka Vágó

**Affiliations:** 1 Heart and Vascular Center, Semmelweis University, Budapest, Hungary; 2 Department of Sports Medicine, Semmelweis University, Budapest, Hungary

**Keywords:** athletes, heart, COVID-19

## Abstract

**Objectives:**

To investigate the cardiovascular consequences of SARS-CoV-2 infection in highly trained, otherwise healthy athletes using cardiac magnetic resonance (CMR) imaging and to compare our results with sex-matched and age-matched athletes and less active controls.

**Methods:**

SARS-CoV-2 infection was diagnosed by PCR on swab tests or serum immunoglobulin G antibody tests prior to a comprehensive CMR examination. The CMR protocol contained sequences to assess structural, functional and tissue-specific data.

**Results:**

One hundred forty-seven athletes (94 male, median 23, IQR 20–28 years) after SARS-CoV-2 infection were included. Overall, 4.7% (n=7) of the athletes had alterations in their CMR as follows: late gadolinium enhancement (LGE) showing a non-ischaemic pattern with or without T2 elevation (n=3), slightly elevated native T1 values with or without elevated T2 values without pathological LGE (n=3) and pericardial involvement (n=1). Only two (1.4%) athletes presented with definite signs of myocarditis. We found pronounced sport adaptation in both athletes after SARS-CoV-2 infection and athlete controls. There was no difference between CMR parameters, including native T1 and T2 mapping, between athletes after SARS-CoV-2 infection and the matched athletic groups. Comparing athletes with different symptom severities showed that athletes with moderate symptoms had slightly greater T1 values than athletes with asymptomatic and mildly symptomatic infections (p<0.05). However, T1 mapping values remained below the cut-off point for most patients.

**Conclusion:**

Among 147 highly trained athletes after SARS-CoV-2 infection, cardiac involvement on CMR showed a modest frequency (4.7%), with definite signs of myocarditis present in only 1.4%. Comparing athletes after SARS-CoV-2 infection and healthy sex-matched and age-matched athletes showed no difference between CMR parameters, including native T1 and T2 values.

## Introduction

The presence and extent of cardiac involvement in patients with COVID-19 are of great interest, especially among highly trained athletes returning to extreme physical activity after the infection. Emerging yet conflicting evidence has led to greater interest in cardiac magnetic resonance (CMR) imaging studies due to its ability to provide tissue-specific information non-invasively. A cohort study by Puntmann *et al*^1^ using late gadolinium enhancement (LGE) and novel T1 and T2 mapping sequences showed myocardial involvement in an alarming 78% of middle-aged patients, raising serious concerns regarding their cardiac health. Approximately one-third of the alterations were solely based on mapping elevations; however, the exact diagnostic and prognostic impact of these contemporary techniques is less well understood than that of widely used techniques such as LGE.^2^

Recently published studies have evaluated cardiac involvement by CMR imaging in athletes who recovered after SARS-CoV-2 infection. Earlier data by Rajpal *et al*^3^ and Brito *et al*^4^ found a high prevalence of myocardial (15%) and pericardial (39.5%) inflammatory alterations among college athletes following SARS-CoV-2 infection. Subsequent publications reported a lower prevalence of cardiac involvement ranging from 0.7% to 3.0% in college athletes after SARS-CoV-2 infection.^5–7^

The most recent expert consensus statements regarding the screening of potential cardiac involvement in competitive athletes recovering from SARS-CoV-2 infection highlight the need for more robust data with the inclusion of appropriate control subjects.^8 9^ Therefore, our study aimed to investigate cardiac involvement after SARS-CoV-2 infection in young competitive athletes using a comprehensive CMR imaging study, including tissue characterisation and feature-tracking strain analysis. We compared our results with those from healthy sex-matched and age-matched athletes and healthy sex-matched and age-matched less active controls.

## Methods

### Study population

**Figure 1:**
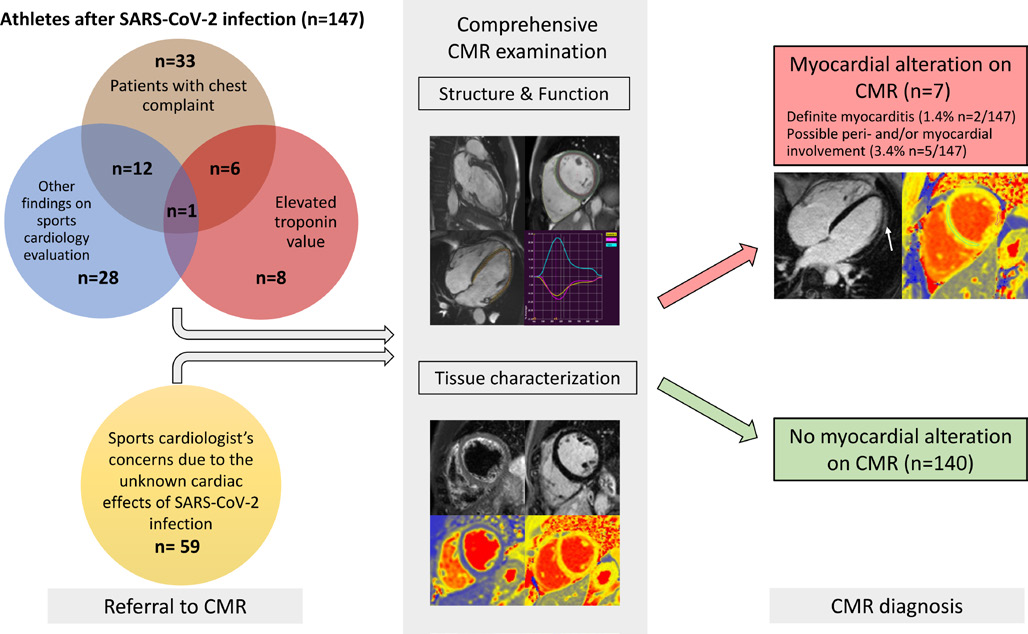
Central illustration. Athletes were referred for CMR by their cardiologists to evaluate the possible structural alterations caused by SARS-CoV-2 infection. CMR referral is summarised as follows: patients who had chest complaints (brown bubble), including chest pain, dyspnoea and palpitation; second, patients who had CMR due to elevated troponin levels (red bubble) with or without accompanying symptoms; third, due to other findings on sports cardiology evaluation (blue bubble) such as alterations on echocardiography and/or 12-lead-ECG examination; lastly, those referred to CMR due to the unknown cardiac effects of the infection (yellow bubble) despite having negative results on cardiology examination. All athletes underwent a comprehensive CMR examination that contained sequences to assess structural, functional (long-axis and short-axis cine images) and tissue-specific data (T2-weighted images, LGE, native T2 and T1 mappings). Overall, we found cardiac involvement on CMR in only seven patients. Only two presented with definite signs of myocarditis (red box, underneath white arrow showing subepicardial LGE). The majority of athletes had no alterations on their CMR (green box). CMR, cardiac magnetic resonance; LGE, late gadolinium enhancement.

All athletes recovering from SARS-CoV-2 infection who were referred to our centre for CMR examination between July 2020 and February 2021 were consecutively included in this observational study (figure 1). SARS-CoV-2 infection was diagnosed by PCR on swab tests or by serum IgG antibody tests prior to CMR examination. We excluded athletes (1) aged <16 years and (2) performing <6 hours of training/week. Athletes were referred for CMR by their cardiologist to evaluate for possible structural alterations caused by SARS-CoV-2 infection, in most cases prior to their return to high levels of sports activity. Detailed information regarding patient referral to CMR is included in figure 1. All athletes completed a sports-specific questionnaire and a questionnaire regarding their SARS-CoV-2-related symptoms. Symptoms were assessed using the COVID-19 treatment guidelines published by the National Institutes of Health.^10^ Asymptomatic SARS-CoV-2 infection was defined for individuals who tested positive for SARS-CoV-2 and had no symptoms consistent with COVID-19. Mild symptoms were defined as symptoms such as fever, cough, headache, loss of smell and/or taste but not more alarming signs, such as chest pain, dyspnoea and shortness of breath, which were categorised as moderate symptoms. Long-COVID-19 symptoms were persistent symptoms, mostly fatigue and palpitations, extending beyond 4 weeks after the initial infection. Data from the first 12 athletes with post-COVID-19 scanned in our institute published in Journal of the American College of Cardiology (JACC) imaging are incorporated in the current publication.^11^

Clinical data, including 12-lead ECG and high-sensitivity troponin T (hsTnT) were recorded a median of 1 day (0–7 days) prior to the CMR examination. The local laboratory cut-off value for detectable hsTnT was >2.99 ng/L and that for elevated hsTnT was >13.99 ng/L. All examinations were performed after an appropriate quarantine period (10 days).

CMR parameters were compared with those of sex-matched and age-matched healthy athletes (n=59) and healthy, less active controls (n=56). All healthy controls were scanned to establish normal values for the less active and athletic population without any suspicion of cardiovascular pathology prior to the COVID-19 pandemic (59%) or in athletes and volunteers who tested seronegative for the disease (41%). Athletes after SARS-CoV-2 infection and healthy control athletes both performed high levels of sport activity, the majority of them being professional athletes competing at national or international levels in mixed or endurance sports disciplines (table 1).^12^ Healthy, less active controls performed <6 hours of sports activity/week. None of the participants reported a history of cardiovascular disease or consumption of illegal drugs. None of the athletes with post-COVID-19 received steroids during their illness.

**Table 1:** Comparison between athletes after SARS-CoV-2 infection, healthy athlete controls and healthy, less active controls

	Athletes after SARS-CoV-2 infection (n=147)	Healthy athletic controls (n=59)	Healthy, less active controls (n=56)	Athletes after SARS-CoV-2 infection versus healthy athletic controls P values	Athletes after SARS-CoV-2 infection versus healthy, less active controls P values
Group characteristics					
Age (years), median (IQR)	23 (20–28)	25 (21–29)	24 (23–28)	0.146	0.062
Sex: female, N (%)	53 (36)	20 (34)	20 (36)	0.771	0.864
Body surface area (m^2^), average ±SD	2±0.2	2±0.3	1.9±0.2	0.413	0.003
Heart rate (beats/min), median (IQR)	60 (53–69)	62 (56–72)	71 (63–84)	0.032	<0.001
Degree of training (hours/week), median (IQRS)	15 (12–22)	19 (15–22)		0.024	
Sport discipline, N (%)				0.077	
Skill	2 (1)	0 (0)			
Power	9 (6)	9 (15)			
Mixed	108 (74)	35 (60)			
Endurance	28 (19)	15 (25)			
Member of a national team, N (%)	87 (60)	52 (91)		<0.001	
Member of an Olympic team, N (%)	17 (12)	15 (26)		0.014	
CMR parameters					
Standard left and right ventricular parameters					
LVEF (%), median (IQR)	57 (54–60)	56 (53–60)	59 (57–62)	0.473	<0.001
LVEDVi (mL/m^2^), median (IQR)	111 (100–123)	111 (102–122)	91 (83–100)	0.523	<0.001
LVESVi (mL/m^2^), median (IQR)	48 (40–55)	47 (43–53)	38 (34–42)	0.52	<0.001
LVSVi (mL/m^2^), median (IQR)	63 (58–69)	64 (58–68)	54 (50–59)	0.685	<0.001
LVMi (g/m^2^) median (IQR)	58 (49–65)	59 (50–73)	47 (39–51)	0.199	<0.001
RVEF (%), median (IQR)	56 (53–59)	55 (52–58)	57 (54–61)	0.14	0.014
RVEDVi (mL/m^2^), median (IQR)	110 (99–121)	113 (103–127)	90 (79–103)	0.119	<0.001
RVESVi (mL/m^2^), median (IQR)	48 (41–55)	50 (44–59)	38 (33–47)	0.055	<0.001
RVSVi (mL/m^2^), median (IQR)	61 (56–67)	63 (57–68)	53 (47–58)	0.229	<0.001
Global left and right ventricular strain					
LV-GLS (%) median (IQR)	−21 (−23 to −19)	−20 (−23 to 19)	−22 (−24 to −20)	0.942	<0.001
LV-GCS (%), average ±SD	−28±4	−28±4	−31±3	0.426	<0.001
LV-GRS (%), median (IQR)	52 (46–60)	50 (45–58)	56 (53–62)	0.609	<0.001
RV-GLS (%), average ±SD	−24±4	−24±3	−25±4	0.691	0.21
Parametric mapping					
T1 mapping (ms), median (IQR)	958 (939–970)	955 (934–973)	972 (960–987)	0.564	<0.001
T2 mapping (ms), median (IQR)	45 (43–46)	44 (43–46)	44 (43–45)	0.196	0.215

CMR, cardiac magnetic resonance; GCS, global circumferential strain; GLS, global longitudinal strain; GRS, global radial strain; LEDVi, left ventricular end diastolic volume index; LV, left ventricular; LVEF, left ventricular ejection fraction; LVESVi, left ventricular end systolic volume index; LVMi, left ventricular mass index; RV, right ventricular; RVEDVi, right ventricular end diastolic volume index; RVEF, right ventricular ejection fraction; RVESVi, right ventricular end systolic volume index; RVMi, right ventricular mass index; SLVi, left ventricular stroke volume index.

### CMR protocol

CMR examinations were performed on a 1.5 T MRI scanner (Magnetom Aera; Siemens Healthcare, Erlangen, Germany). A comprehensive CMR protocol was carried out, including cine movies, T2-weighted spectral presaturation with inversion recovery, T2 mapping using T2-prep balanced steady-state free precession (b-SSFP), T1 mapping using long-T1 5(3)3 and short-T1 5(3)3 modified look-locker inversion recovery and LGE imaging. Functional imaging was performed using b-SSFP cine sequences in four-chamber, two-chamber and three-chamber long-axis views and a short-axis (SA) stack from the cardiac base to apex with full coverage of the left ventricle (LV) and th right ventricle (RV). Overall, 139 athletes after SARS-CoV-2 infection and 15 healthy control athletes agreed to receive contrast agent. None of the healthy, less active controls were given contrast material. LGE images were acquired using a segmented inversion recovery sequence 10–15 min after the administration of an intravenous bolus of 0.15 mmol/kg gadolinium-based contrast agent gadobutrol (Gadovist, Bayer-Schering Pharma) at a rate of 2–3 mL/s through an antecubital intravenous line. The inversion time was adjusted to provide optimal suppression of normal myocardium.

### Image analysis

All postprocessing analyses were performed using Medis Suite Software (Medis Medical Imaging Software, The Netherlands). LV and RV volumes, function and mass were calculated from the SA stack using artificial intelligence–based automated contour detection (autoQ application) with manual adjustments if required. Myocardial native T1 and T2 relaxation times were measured conservatively in the midventricular or basal septum (if the midventricular images were technically inadequate for analysis) of the myocardium using motion-corrected images^13^ by an experienced observer blinded to the clinical data and group of a given subject. In case of suspicion of focal T1 mapping elevation, a separate region of interest in that area was drawn. Quantitative deformation assessment was obtained using cine images and analysed using the QStrain application. Global strain values, including LV longitudinal (global longitudinal strain (GLS)), circumferential, radial and RV longitudinal, and free wall strain, were measured. The interpretation of LGE was standardised as follows: myocardial and pericardial LGE was visually defined by two observers based on the presence and pattern. All images were visually assessed by two observers blinded to the clinical data of a given subject. In case of disagreement between the observers, a third CMR specialist with an European Association of Cardiovascular Imaging level 3 certificate was consulted for consensus. Non-ischaemic LGE was defined as midmyocardial and/or subepicardial myocardial LGE confirmed in two perpendicular views. Pericardial involvement was reported if the pericardium showed definite LGE and the thickness of the pericardium was >2 mm regardless of pericardial oedema. Hinge point fibrosis was defined as a small volume of focal LGE confined to the inferoseptal segment, where the RV attaches to the septum. We classified cardiac involvement as definite in case of CMR T1 abnormality or LGE showing pathological pattern and CMR T2 abnormality and one or more supporting findings such as decreased LV ejection fraction or elevated troponin level. Possible pericardial/myocardial involvement was reported when we found (1) mild CMR T1 abnormality or the presence of LGE with normal T2, or (2) mildly elevated T1 and T2 mapping with no LGE or other supporting findings.

### Follow-up

We performed midterm follow-up using the institutional electronic database for the patients who returned to our clinic, and we contacted the other athletes via telephone. Athletes completed a questionnaire regarding any ongoing symptoms, their ability to return to high sports activity levels, and their overall experience during the CMR examination. We offered a follow-up cardiological examination, including a CMR scan at our institution, to all athletes reporting reinfection with SARS-CoV-2. All athletes with definite or possible myocardial alteration on their baseline scan were contacted and offered a follow-up CMR examination.

### Data management and statistical analysis

The Shapiro-Wilk test was applied to test the normality of our data. Continuous variables showing a normal distribution are presented as the mean and SD, and those showing a non-normal distribution are reported as medians and IQRs. Categorical variables are presented as frequencies and percentages. Comparisons between participant groups were conducted using independent samples t-tests and Mann-Whitney U tests as appropriate. Non-normal continuous variables were compared by the Kruskal-Wallis test. χ^2^ tests were applied to compare the distributions of categorical data. Associations were assessed using Spearman’s rank correlation analyses. Probability values were two-sided, and p values of <0.05 were considered significant. Elevated T1 and T2 values were defined based on the sequence-specific cut-offs of 2 SDs above the respective means of the healthy, sex-matched and age-matched athlete controls (male athletes: T1: 986 ms, T2: 46 ms; female athletes: T1: 1001 ms, T2: 49 ms). MedCalc software V.18.11 (Belgium) and RStudio V.1.3.1.093 (RFoundation, Austria) were used for statistical analysis and graph generation. All data are available on reasonable request.

## Results

Overall, 147 (94 male, median 23, IQR 20–28 years) athletes with prior SARS-CoV-2 infection were included in our study. They were asymptomatic (n=19) or experienced mild (n=80), moderate (n=43) or long-COVID-19 (n=5) symptoms, and none of them required hospital treatment. CMR imaging was performed at a median of 32 days after a positive PCR test. Overall, 4.7% (n=7) of patients had alterations in their CMR scans, and none of these athletes were asymptomatic. The CMR findings were as follows: LGE showing a non-ischaemic pattern and elevated native T1 mapping consistent with acute myocarditis as per the Lake Louise criteria (n=1); LGE showing a nonischaemic pattern consistent with previous myocarditis with only mildly elevated T2 values (n=1); non-specific nonischaemic LGE (n=1); slightly elevated T1 and T2 values with no pathological LGE (n=2); isolated, slightly elevated T1 value (n=1); and pericardial involvement (n=1). All athletes with definite (n=2) or possible (n=5) myocardial or pericardial alterations were referred to CMR examination based on the clinical suspicion of myocardial involvement as detailed in table 2. HsTnT recorded in our institute was elevated in 4.5% of the cases (n=6/133); among these patients, only one had myocardial alteration on CMR.

**Table 2:** Detailed information regarding athetes with post-COVID-19 with myocardial or pericardial alterations on cardiac MRI

Athlete no	Sex	Symptoms	Findings on other exams	Time to CMR after positive test results (days)	hsTnT recorded prior to CMR (ng/L)	CMR findings	Pathological alteration	Certainty of cardiac involvement	Clinical outcome (6 months)
1.	Male	Moderate Chest pain. Fever. Headache. Joint pain. Diarrhoea. Smell and taste disturbance.	Troponin: elevated (hsTnT: 18 ng/L, normal: <14 ng/L) 12-lead ECG: minor repolarisation. alteration Holter ECG: sinus tachycardia (1 hour) Echocardiography: slightly dilated RV Exercise test (4 months after COVID-19 infection): normal	70	18	LVEF: 52% GLS: −18% Septal native T1: normal Septal native T2: normal Pathological LGE/pattern: yes—lateral subepicardial T1 and T2 mapping value in the area corresponding with the LGE: 1016 and 50 ms—mildly elevated	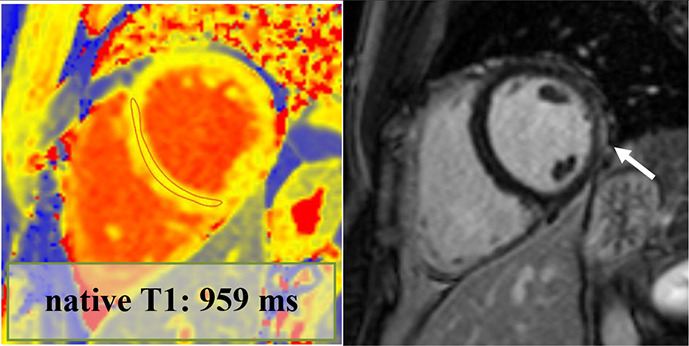	Definite	Returned to sport, no persistent cardiac complaints at follow-up
2.	Male	Moderate Chest pain. Dyspnoea. Fever. Cough.	Troponin: elevated (hs troponin I: 198 ng/L, normal: <45 ng/L) 12-lead ECG: minor repol. alteration Holter ECG: normal Echocardiography: normal Exercise test (3 months after COVID-19 infection): normal	74	NA	LVEF: 58% GLS: −18% Septal native T1: elevated Septal native T2: normal Pathological LGE/pattern: Yes—lateral subepicardial T1 and T2 mapping value in the area corresponding with the LGE: 1065 and 53 ms—elevated	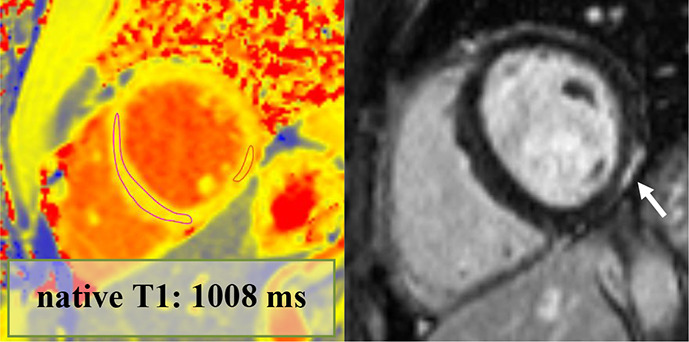	Definite	Returned to sport, no persistent cardiac complaints at follow-up
3.	Male	Moderate Chest pain. Dyspnoea. Fatigue. Cough.	Troponin: normal 12-lead ECG: RBBB (previously reported) Holter ECG: not performed Echocardiography: normal Exercise test (5 months after COVID-19 infection): normal	27	4	LVEF: 61% GLS: −22% Septal native T1: normal Septal native T2: normal Pathological LGE/pattern: Yes—non-specific inferior and hinge point LGE T1 and T2 mapping value in the area corresponding with the LGE: 984 and 41 ms—normal	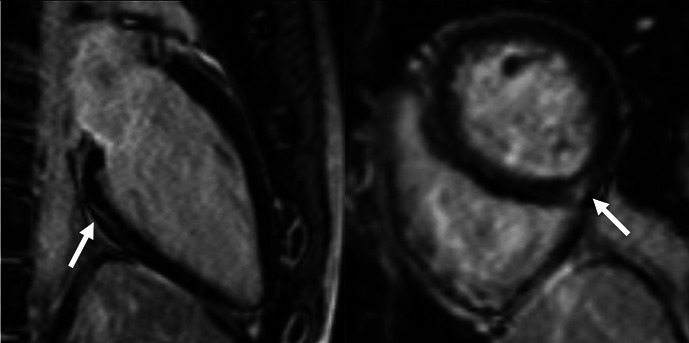	Possible	Returned to sport, no persistent cardiac complaints at follow-up
4.	Female	Long COVID-19 Palpitation. Long-lasting fatigue.	Troponin: normal 12-lead ECG: normal Holter ECG: normal Echocardiography: normal Exercise test (5 months after COVID-19 infection): normal	67	<3	LVEF: 67% GLS: −27% Septal native T1: grey zone normal/elevated Septal native T2: mildly elevated Pathological LGE/pattern: no	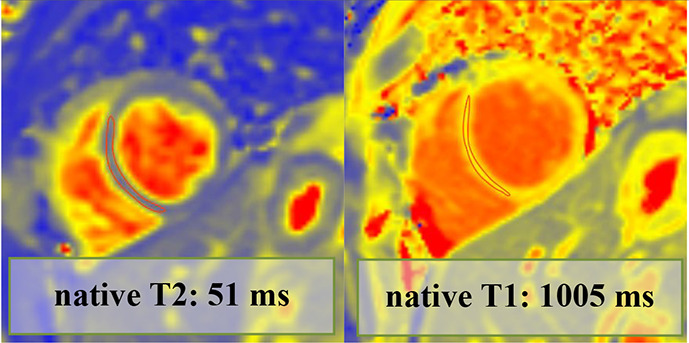	Possible	Returned to sport, no persistent cardiac complaints at follow-up
5.	Female	Moderate Chest pain. Back pain. Smell and taste disturbance.	Troponin: normal 12-lead ECG: PVC Holter ECG: trigeminy PVC on exertion Echocardiography: normal Exercise test (4 months after COVID-19 infection): normal	19	<3	LVEF: 60% GLS: −22% Septal native T1: mildly elevated Septal native T2: mildly elevated Pathological LGE/pattern: No	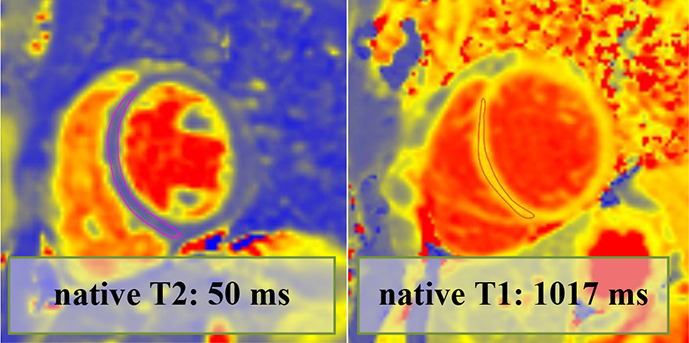	Possible	Returned to sport, no ongoing cardiac complaints.
6.	Female	Mild Fever. Fatigue. Palpitation. Smell and taste disturbance.	Troponin: elevated (hs troponin I: 28 ng/L—normal: <1.9 ng/L) 12-lead normal Holter ECG: NA Echocardiography: normal Exercise test: NA	11	<3	LVEF: 55% GLS: −18% Septal native T1: mildly elevated Septal native T2: normal Pathological LGE/pattern: no	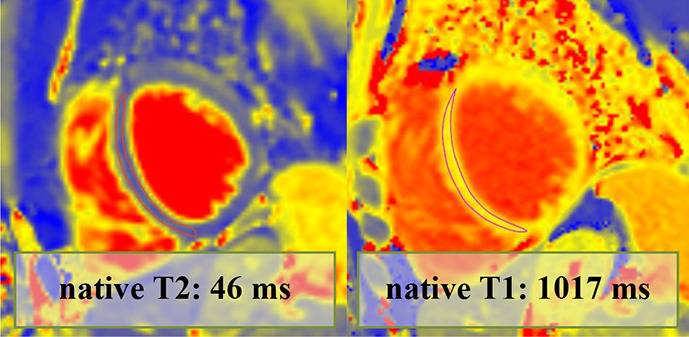	Possible	Returned to sport, no ongoing cardiac complaints
7.	Male	Moderate Chest pain. Long-lasting fatigue.	Troponin: elevated (hs troponin I: 225 ng/L—normal: <45 ng/L) 12-lead ECG: descending PQ segment depression Holter ECG: NA Echocardiography: decreased longitudinal strain, mild anterior and anteroseptal wall motion abnormality Holter ECG: NA	120	10	LVEF: 61% GLS: −20% Septal native T1: normal Septal native T2: normal Pathological LGE/pattern: Yes—pericardial involvement	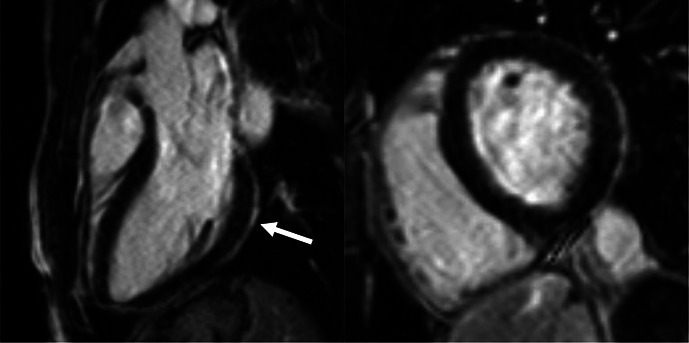	Possible	Returned to sport, no ongoing cardiac complaints

CMR, cardiac magnetic resonance; GLS, global longitudinal strain; hsTnT, high-sensitivity troponin T; LVEF, left ventricular ejection fraction; NA, not applicable; PVC, premature ventricular complex.

We found hinge point fibrosis in 32% (n=44) of the athletes after SARS-CoV-2 infection, which we reported as non-pathological. Fifteen healthy control athletes received contrast material. The proportion of hinge point fibrosis was similar in athletes after SARS-CoV-2 infection (44/139, 32%) and healthy control athletes (6/15, 40%; p=0.513).

Table 1 shows the comparison between highly trained athletes with prior SARS-CoV-2 infection, healthy athletic controls and healthy less active controls. We found elevated cardiac volumes and myocardial mass in athletes relative to less active controls, signifying normal sport adaptation. There were no differences between the matched athletic groups regarding their LV and RV functional and volumetric parameters. LV analysis showed subtle functional alterations between athletes and controls, with the former showing slightly lower strain values. There was no difference regarding any strain parameters between athletes after SARS-CoV-2 infection and healthy control athletes. Native T1 values were slightly lower in the athletes after SARS-CoV-2 infection than in the controls, but there was no difference between athletic groups. The T2 values were not different among the three groups.

**Figure 2:**
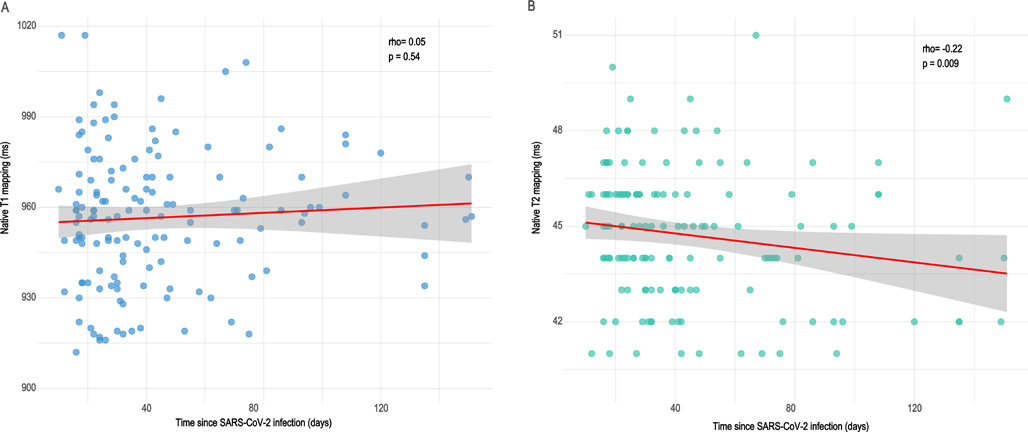
Associations of native T1 and T2 mapping values and the time from confirmation of SARS-CoV-2 infection. We did not find a correlation between T1 values and time since SARS-CoV-2 infection, while T2 values showed a weak negative correlation with this parameter.

We explored the associations of native T1 and T2 mapping values with the time since confirmation of SARS-CoV-2 infection (figure 2). We did not find a correlation between T1 values and time since the infection, while T2 values showed a weak negative correlation (Rho: −0.22, p=0.009) with this parameter.

Comparison of native T1 mapping values between sexes revealed that men (median 953, IQR 934–965 ms) had significantly lower T1 values than women (median 977, IQR 959–987 ms), regardless of whether they were healthy controls or athletes after SARS-CoV-2 infection (p<0.0001) (online supplemental file 1).

10.1136/bjsports-2021-104576.supp1 Supplementary data

Fourteen elite athletes had previously undergone CMR imaging in our institute prior to obtaining positive SARS-CoV-2 PCR results (table 3). The two CMR scans for this group were performed an average of 384 days apart. Comparing examinations before and after the infection revealed no differences regarding any CMR parameters, as shown in table 3.

**Table 3:** Comparison between CMR examinations before and after SARS-CoV-2 infection

	CMR scan before SARS-CoV-2 infection (n=14)	CMR scan after SARS-CoV-2 infection (n=14)	P values
Standard left and right ventricular CMR parameters			
LVEF (%), median (IQR)	55 (53–58)	57 (53–61)	0.091
LVEDVi (mL/m^2^), median (IQR)	111 (103–120)	117 (104–125)	0.305
LVESVi (mL/m^2^), median (IQR)	47 (46–59)	51 (42–55)	0.216
LVSVi (mL/m^2^), median (IQR)	65 (57–67)	65 (60–75)	0.135
LVMi (g/m^2^), median (IQR)	63 (59–77)	70 (62–82)	0.502
RVEF (%), median (IQR)	54 (52–56)	57 (53–60)	0.091
RVEDVi (mL/m^2^), median (IQR)	113 (107–120)	116 (100–122)	0.946
RVESVi (mL/m^2^), median (IQR)	53 (44–60)	49 (45–57)	0.094
RVSVi (mL/m^2^), median (IQR)	62 (57–69)	64 (59–73)	0.38
Global left and right ventricular strain			
LV-GLS (%), median (IQR)	−20 (−22 to −19)	−20 (−21 to −18)	0.241
LV-GCS (%), average ±SD	−27±3	−28±5	0.883
LV-GRS (%), median (IQR)	50 (45–55)	49 (45–53)	0.715
RV-GLS (%), average ±SD	−24±3	−23±3	0.29
Parametric mapping			
T1 mapping, median (IQR), ms	947 (932–961)	937 (933–966)	0.791
T2 mapping, median (IQR), ms	43 (43–45)	44 (42–46)	0.32

CMR, cardiac magnetic resonance; GCS, global circumferential strain; GLS, global longitudinal strain; GRS, global radial strain; LV, left ventricular; LVEDVi, left ventricular end diastolic volume index; LVEF, left ventricular ejection fraction; LVESVi, left ventricular end systolic volume index; LVMi, left ventricular mass index; LVSVi, left ventricular stroke volume index; RV, right ventricular; RVEDVi, right ventricular end diastolic volume index; RVEF, right ventricular ejection fraction; RVESVi, right ventricular end systolic volume index; RVSVi, right ventricular stroke volume index.

**Figure 3:**
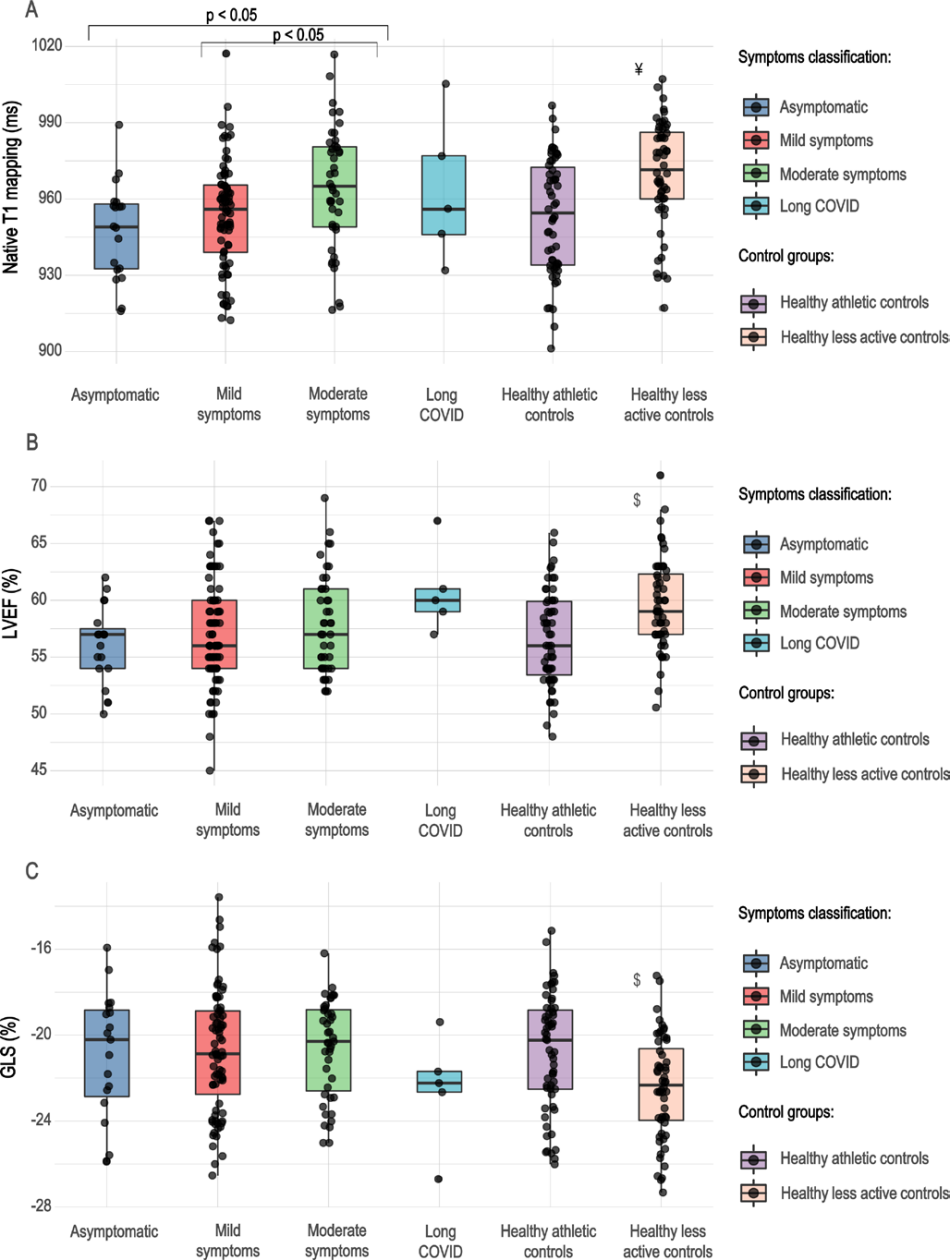
Boxplots of native T1 mapping, LVEF and GLS values by symptom group. Moderately symptomatic athletes with post-COVID-19 had elevated native T1 values relative to asymptomatic and mildly symptomatic infections (p<0.05). However, the T1 value remained below the normal cut-off point for the majority of patients. There was no difference in the LVEF or GLS values among these groups. ¥, Kruskal-Wallis test showing a significant difference between healthy, less active controls and asymptomatic and mildly symptomatic athletes after SARS-CoV-2 infection and healthy athletic controls; $, Kruskal-Wallis test showing a significant difference between healthy, less active controls and asymptomatic, mildly and moderately symptomatic athletes after SARS-CoV-2 infection, and healthy athletic controls. GLS, global longitudinal strain; LVEF, left ventricular ejection fraction.

We compared athletes with prior SARS-CoV-2 infection regarding their symptoms (figure 3), which showed that athletes with moderate symptoms, mainly chest pain and dyspnoea, had slightly elevated native T1 values relative to their asymptomatic and mildly symptomatic counterparts (p<0.05). However, the T1 value remained below the cut-off point for the majority of patients. Furthermore, there was no difference in the LV ejection fraction or GLS values among these groups.

We obtained follow-up in 122 (83%) athletes after SARS-CoV-2 infection at a median of 232 days after the infection. All but two athletes could return to sports activity safely. One of them did not return to sports due to the progression of his depression, and he currently receives medication. The other athlete experienced long-COVID syndrome, including light-headedness and long-term rapid increase in his heartbeat. At the time of our follow-up, this athlete had a negative exercise test and was advised to restart sports activity. The outcomes of the seven athletes with CMR alteration are shown in table 2.

Online supplemental file 2 shows the acute and follow-up CMR scans in those patients with myocardial alteration (n=4) who returned for a follow-up scan. In one athlete with LGE showing a non-ischaemic pattern consistent with previous myocarditis, the follow-up CMR showed slightly elevated systolic function and the shrinkage of the LGE. Among the three patients presenting with mild, isolated mapping elevation, the follow-up scan revealed that the elevated mapping values had subsided for two patients and remained slightly elevated for the last. Three athletes asked to postpone their follow-up scans due to their lack of symptoms and their ongoing sports season.

10.1136/bjsports-2021-104576.supp2 Supplementary data

Overall, 10 athletes reported a subjectively long recovery from COVID-19. Three additional athletes said that, although they returned to sports activity, they did not reach their peak potential at the time of their follow-up. It was due to anxiety in one case and two athletes experienced mild, long-term sinus tachycardia with no apparent structural alteration. None of the national team members (n=71) reported significant setbacks in their performance. In all patients who reported reinfection confirmed by PCR (n=4), we performed follow-up CMR without definite alteration (online supplemental file 3).

10.1136/bjsports-2021-104576.supp3 Supplementary data

## Discussion

The current study presents a comprehensive analysis of the CMR findings of 147 highly trained athletes following SARS-CoV-2 infection and compares them to sex-matched and age-matched healthy athletes and less active controls. In this group, where all athletes were referred to the examination by a cardiologist, CMR revealed no overall differences regarding any volumetric, functional or tissue characteristics between athletes with prior SARS-CoV-2 infection and matched healthy athletes. However, a minority of the athletes had definite (n=2, 1.4%) or possible (n=5, 3.4%) myocardial or pericardial alterations on CMR. Four of these athletes were moderately symptomatic; two of them had long COVID; and one had mild symptoms.

Among young highly trained athletes, we found a lower frequency of myocardial alteration than previously reported by Rajpal and colleagues,^3^ who performed CMR for 26 asymptomatic or mildly symptomatic collage athletes with negative troponin levels and normal ECG and echocardiography. They found that 46% of the athletes had LGE and 15% had myocardial alterations interpreted as acute myocarditis. In our study, only one patient had CMR findings consistent with acute myocarditis as per the Lake Louise criteria,^14^ and one had findings suggesting previous myocarditis. As per those three athletes who presented with slightly elevated T1 values with or without elevated T2 values, we reported possible mild diffuse myocardial involvement and performed a follow-up CMR scan, which showed the resolution of these alterations in two patients. Our results are quite similar to those found by Starekova *et al*, Moulson *et al* and Martinez *et al*,^5 7 15^ signifying the modest prevalence of myocardial involvement after SARS-CoV-2 in young, otherwise healthy individuals. In a nationwide research study among US collegiate athletes conducted by Moulson and colleagues, they also found the cardiac involvement among athletes as low as 0.7%, and interestingly, they found that CMR scans performed on the basis of clinical symptoms were four times more likely to show myocardial alterations as opposed to those that were performed as a primary screening method.^7^ Overall, these findings are in line with pathological reports showing that only 1%–7% of 277 autopsied hearts across 22 publications had COVID-19-related myocarditis according to histopathological findings,^16^ although in a different patient population. In our cohort, only one athlete presented with pericardial involvement; this finding is in contrast with the case series of Brito and colleagues, who found pericardial enhancement in 39.5% of athletes.^4^

We did not find a difference regarding the proportion of hinge point fibrosis after SARS-CoV-2 infection in athletes and healthy control athletes; however, only a relatively small number of control athletes received contrast material (n=15). We found a slightly higher proportion of hinge point fibrosis than Clark *et al* (athletes after SARS-CoV-2 infection) and a lower ratio than Domenech-Ximenos *et al* in endurance athletes before the pandemic (32% vs 22% vs 38%). Of note, these athletic groups were different from ours in some respects, including the ratio of female athletes (36% vs 63% vs 47%), sports discipline and training hours, which might account for the differences.^6 17^

Our findings regarding sport adaptation are in line with the current literature.^18 19^ Data are scarce regarding the feature-tracking strain analysis of highly trained athletes, and the tendencies described in our study (slightly lower global strain values among highly trained men) are similar to those in the currently available publications using echocardiography.^20–22^ Comparing athletes with prior SARS-CoV-2 infection and matched athletes showed no difference between CMR parameters, including strain parameters and native T1 and T2 mapping values. This finding confirms the results of the recent research letter by Clark *et al*,^6^ who reported only a small difference between athletes post-COVID-19 and healthy control athletes regarding their mid-septal T2 mapping values. However, the groups in their study were matched by training load, not age or sex, which could have contributed to differences. While the cohort study by Puntmann *et al*^1^ reported a higher prevalence of findings, new studies have shown similar results to ours, although in very different populations.^23 24^ McDiarmid *et al*^25^ previously demonstrated that physiological hypertrophy slightly decreased the T1 value among highly trained athletes. We also found that, similar to other CMR parameters, men and women have distinct native T1 and T2 values, which justifies the use of sex-matched control groups when interpreting mapping alterations.

We did not find a correlation between T1 mapping values and the time passed since SARS-CoV-2 infection, similar to what Knight *et al*^24^ found in their study with a somewhat longer delay between SARS-CoV-2 infection and CMR examination (median 68 vs 32 days). A weak but significant correlation was found between T2 mapping and time since infection. This might suggest a reduction in subclinical oedema over time; however, we need more information to confirm this finding.

One unique strength of this study is that 14 athletes had undergone a previous CMR scan at our institute with a standardised protocol; therefore, we were able to compare the results of the two scans. This comparison, however, showed no differences between CMR parameters before and after SARS-CoV-2 infection.

Follow-up at median 232 days after COVID-19 infections showed the majority of athletes returned to high levels of sports activity (n=120/122), although some could not reach their peak performance (n=3) and some experienced reinfection (n=4).

The comparison between athletes with different symptoms revealed slightly elevated T1 mapping values among athletes with chest complaints relative to asymptomatic and mildly symptomatic athletes; however, this did not lead to a reduction in systolic heart function. Moreover, T1 values remained in the normal range for most patients. Currently, there are no data regarding the subclinical cardiac alterations caused by mild forms of systemic viral infections such as influenza and whether they are detectable on CMR. We believe that studies investigating the long-term impact of isolated T1 and T2 mapping elevations are necessary to understand the exact prognostic significance of these alterations, and in this study, we share the concerns of Moulson and Baggish^26^ regarding the use of these highly sensitive, although less well-understood techniques, in the screening of otherwise healthy athletes with prior SARS-CoV-2 infection. The current consensus document^9^ regarding the use of CMR in athletes after SARS-CoV-2 infection highlights the importance of well-established screening methods such as troponin, ECG and echocardiography. Moreover, in suspected arrhythmias, further examinations such as 24-hour Holter monitoring might be beneficial,^8 9^ and premature ventricular beats on exercise test might suggest scar on CMR examination as demonstrated by recent studies, enabling a better targeting of CMR scans.^27^ In agreement with this, our results caution against the routine use of CMR for troponin-negative, asymptomatic, or mildly symptomatic patients with COVID-19, as it may lead to false conclusions.

## Limitations

This was a single-centre study performed in a major CMR referral centre. Approximately one-third of the athletes after SARS-CoV-2 infection were referred from other institutions; therefore, their clinical data were provided by the referring clinicians. All athletes included in our study were Caucasian and experienced asymptomatic, mild/moderate or long COVID-19; thus, our conclusions are only applicable to this specific group. Because our study included patients referred by a cardiologist, the reported prevalence of abnormal CMR findings may be overestimated compared with a non-selected population of athletes with SARS-CoV-2 infection. In addition, the clinical implications of CMR abnormalities in the absence of cardiovascular symptoms remains unknown. Lastly, only a proportion of healthy control athletes received contrast agent during their CMR; thus, findings related to LGE in the athletic control group could have been missed.

## Conclusion

Among 147 consecutively included highly trained athletes after SARS-CoV-2 infection and referred by a cardiologist, we found cardiac involvement in 4.7% using CMR, among whom only two (1.4%) presented with definite signs of myocarditis. Our results suggest that cardiac involvement occurs with modest frequency among asymptomatic and mildly/moderately symptomatic SARS-CoV-2 infections in young athletes. Comparisons between athletes after SARS-CoV-2 infection and matched healthy athletes showed no difference between CMR parameters, including strain parameters and T1 and T2 mapping values. Moreover, there was no difference in CMR parameters among athletes examined before and after the infection. The follow-up revealed that the majority of athletes returned to high levels of sports activity without any persisting symptoms.

**What are the new findings?**

- Among 147 highly trained athletes after SARS-CoV-2 infection, we found that cardiac involvement on cardiac magnetic resonance (CMR) was present in only seven (4.8%) patients, among whom only two (1.4%) presented with definite signs of myocarditis.
- Comparing athletes after SARS-CoV-2 infection and healthy sex-matched and age-matched athletes showed no difference between CMR parameters, including strain and native T1 and T2 mapping values.
- Comparison between CMR examinations before and after the infection (n=14) revealed no differences regarding any CMR parameters.
- Follow-up at a median of 232 days after the infections showed the majority of athletes returned to high levels of sports activity (n=120/122, 98.4%).

**How might it impact on clinical practice in the future?**

- Cardiac involvement has a low prevalence among highly trained athletes after SARS-CoV-2 infection.
- Matched control groups are essential for the interpretation of isolated T1 or T2 mapping alterations.
- Our results caution against the routine use of CMR for troponin-negative, asymptomatic or mildly symptomatic patients after SARS-CoV-2 infection.

## Data Availability

Data are available upon reasonable request. All data are available upon reasonable request.
