# Supplementary figures and images for: Is cardiac involvement prevalent in highly trained athletes after SARS-CoV-2 infection? A cardiac magnetic resonance study using sex-matched and age-matched controls
Source: Br J Sports Med. 2021 Nov 30;56(10):553–60. doi: 10.1136/bjsports-2021-104576 (PMC8637606; doi:10.1136/bjsports-2021-104576)

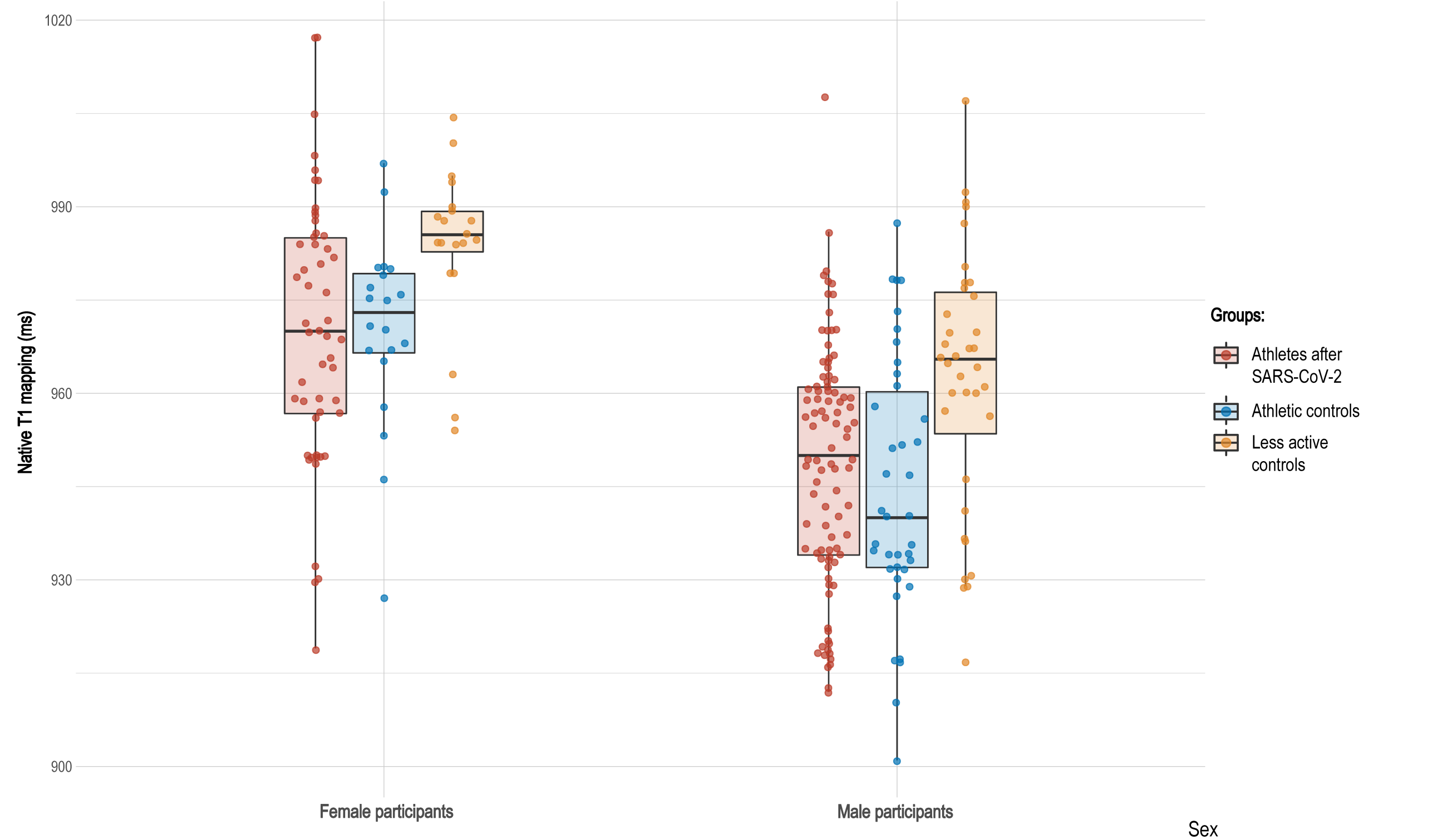

Supplement: Supplementary data [file bjsports-2021-104576supp001.pdf]
