# Supplementary material for: Is cardiac involvement prevalent in highly trained athletes after SARS-CoV-2 infection? A cardiac magnetic resonance study using sex-matched and age-matched controls
Source: Br J Sports Med. 2021 Nov 30;56(10):553–60. doi: 10.1136/bjsports-2021-104576 (PMC8637606; doi:10.1136/bjsports-2021-104576)
Supplement: Supplementary data [file bjsports-2021-104576supp003.pdf]

Supplement table 3 - CMR findings of post-COVID athletes with PCR confirmed reinfection

Athlete No.	Sex	Symptoms at the first infection	CMR findings	CMR images	Symptoms at the reinfection	Time to follow-up CMR from COVID-19 (days)	Follow-up CMR findings	Follow-up CMR images
8.	Male	Asymptomatic	LVEF: 51 %<br>GLS: -19 %<br>LVEDVi: 111 ml/m^2<br>LVMi: 60 g/m^2<br><br>Septal native T1: normal<br>Septal native T2: normal<br>Pathological LGE / pattern: No	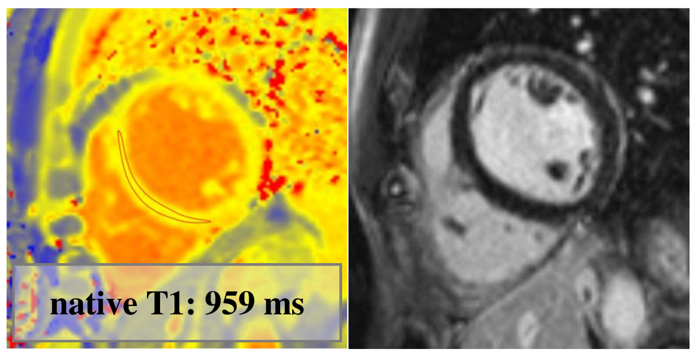	Mild<br>• fever<br>• fatigue<br>• smell and taste disturbance	92	LVEF: 55 %<br>GLS: -18 %<br>LVEDVi: 114 ml/m^2<br>LVMi: 64 g/m^2<br><br>Septal native T1: normal<br>Septal native T2: normal<br>Pathological LGE / pattern: No	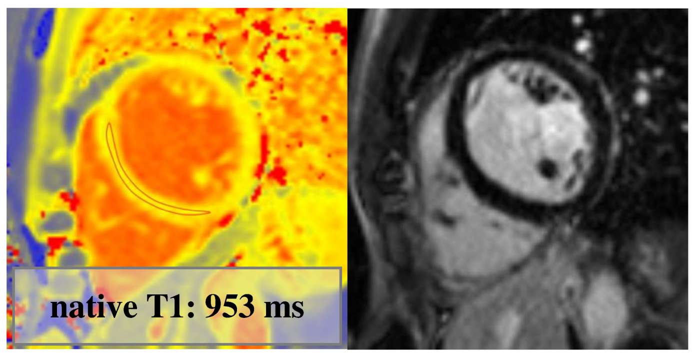
9.	Male	Asymptomatic	LVEF: 57 %<br>GLS: -23 %<br>LVEDVi: 131 ml/m^2<br>LVMi: 67 g/m^2<br><br>Septal native T1: normal<br>Septal native T2: normal<br>Pathological LGE / pattern: No	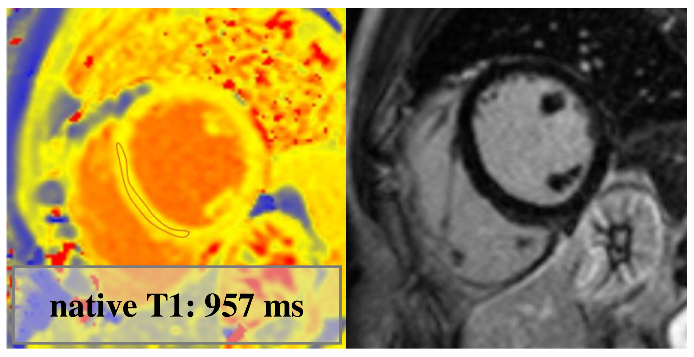	Mild<br>• fever<br>• headache<br>• fatigue<br>• smell and taste disturbance	91	LVEF: 56 %<br>GLS: -20 %<br>LVEDVi: 129 ml/m^2<br>LVMi: 62 g/m^2<br><br>Septal native T1: normal<br>Septal native T2: normal<br>Pathological LGE / pattern: No	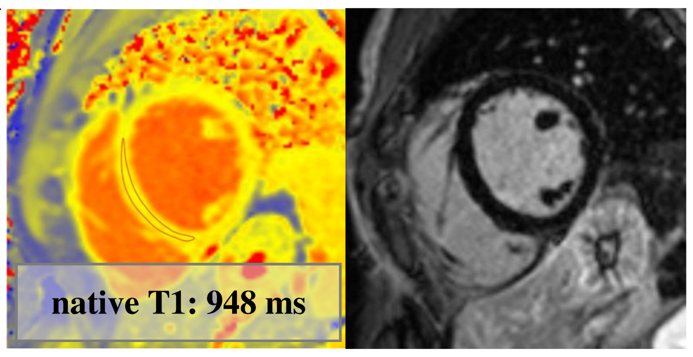
10.	Male	Mild<br>• fatigue<br>• smell and taste disturbance	LVEF: 54 %<br>GLS: -22 %<br>LVEDVi: 144 ml/m^2<br>LVMi: 74 g/m^2<br><br>Septal native T1: normal<br>Septal native T2: normal<br>Pathological LGE / pattern: No	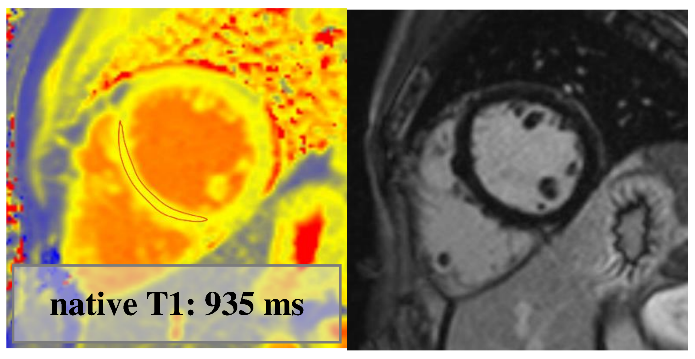	Mild<br>• fever<br>• fatigue<br>• smell and taste disturbance	92	LVEF: 57 %<br>GLS: -20 %<br>LVEDVi: 133 ml/m<br>LVMi: 71 g/m^2<br><br>Septal native T1: normal<br>Septal native T2: normal<br>Pathological LGE / pattern: No	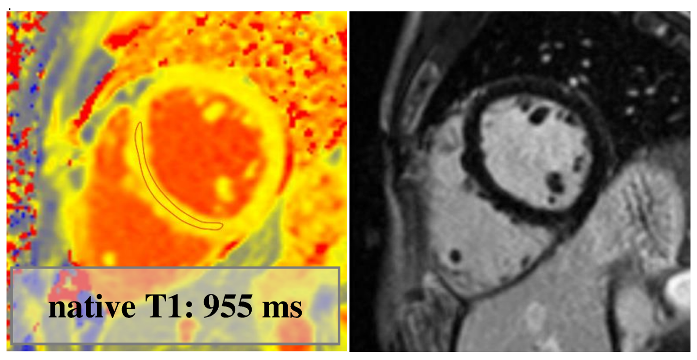
11.	Male	Asymptomatic	LVEF: 55 %<br>GLS: -18 %<br>LVEDVi: 125 ml/m^2<br>LVMi: 70 g/m^2<br><br>Septal native T1: normal<br>Septal native T2: normal<br>Pathological LGE / pattern: No	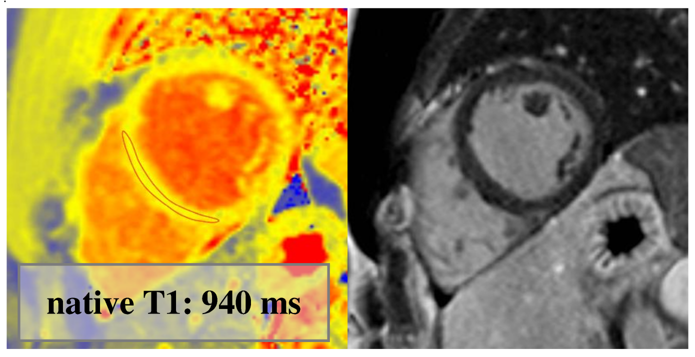	Moderate<br>• headache<br>• fever<br>• mild chest pain<br>• cough<br>• smell and taste disturbance	184	LVEF: 56 %<br>GLS: -18 %<br>LVEDVi: 121 ml/m2<br>LVMi: 62 g/m2<br><br>Septal native T1: normal<br>Septal native T2: normal<br>Pathological LGE / pattern: No	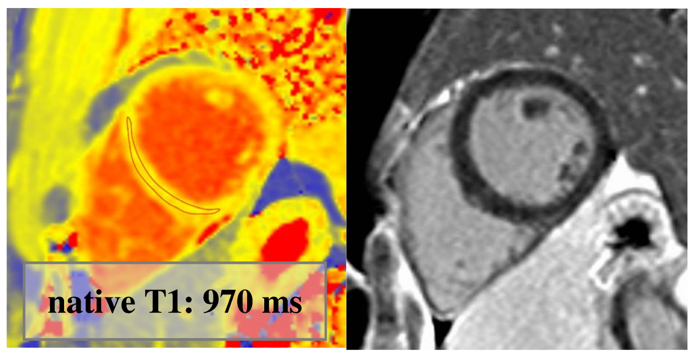
