# Supplementary material for: Is cardiac involvement prevalent in highly trained athletes after SARS-CoV-2 infection? A cardiac magnetic resonance study using sex-matched and age-matched controls
Source: Br J Sports Med. 2021 Nov 29:bjsports-2021-104576. doi: 10.1136/bjsports-2021-104576 (PMC8637606; doi:10.1136/bjsports-2021-104576)
Supplement: Supplementary file 1 [file bjsports-2021-104576supp001.pdf]

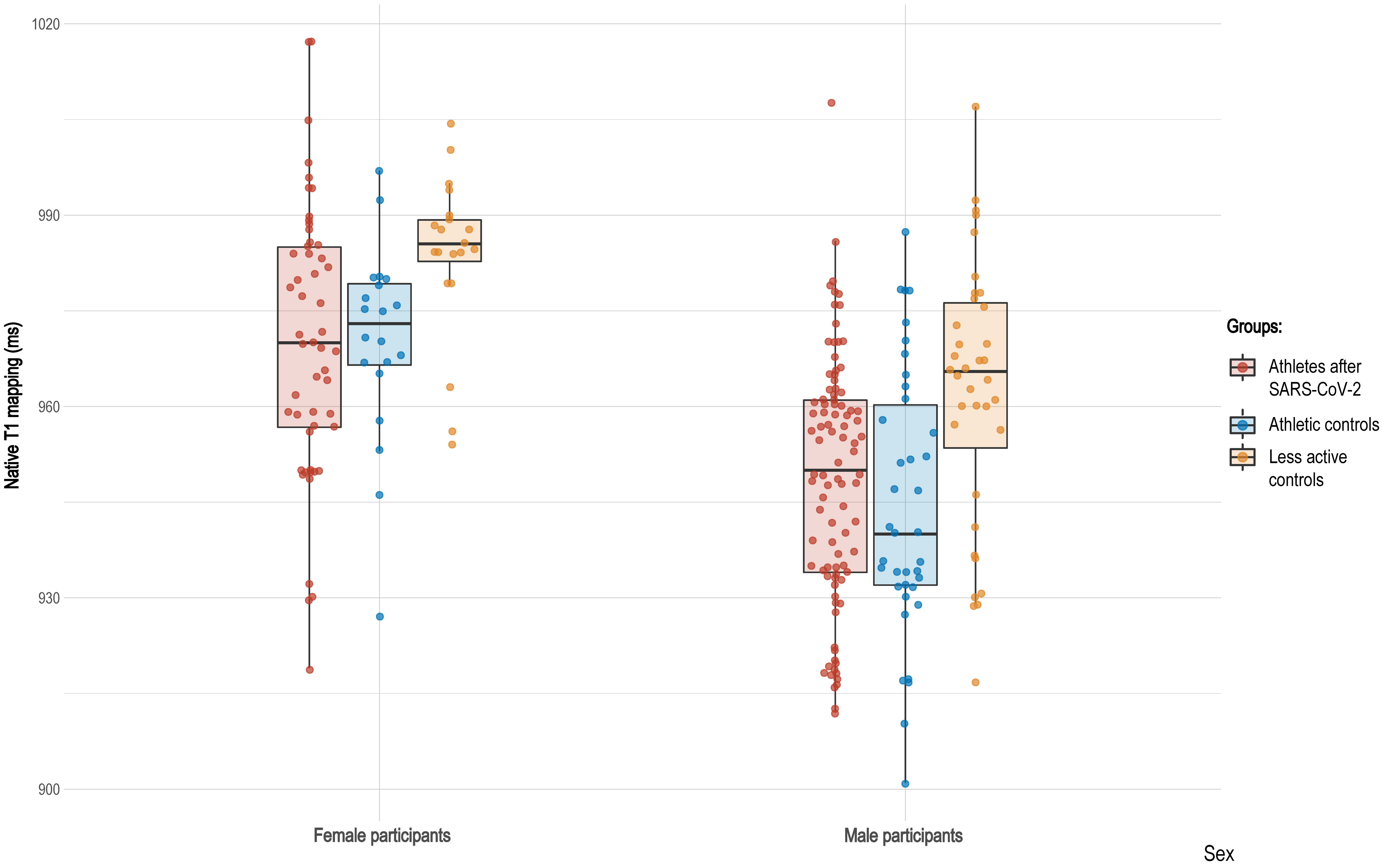
Native T1 mapping (ms)
1020
990
960
930
900
Female participants
Male participants
Sex
Groups:
Athletes after SARS-CoV-2
Athletic controls
Less active controls
