# Supplementary material for: Is cardiac involvement prevalent in highly trained athletes after SARS-CoV-2 infection? A cardiac magnetic resonance study using sex-matched and age-matched controls
Source: Br J Sports Med. 2021 Nov 29:bjsports-2021-104576. doi: 10.1136/bjsports-2021-104576 (PMC8637606; doi:10.1136/bjsports-2021-104576)
Supplement: Supplementary file 2 [file bjsports-2021-104576supp002.pdf]

BMJ Publishing Group Limited (BMJ) disclaims all liability and responsibility arising from any reliance placed on this supplemental material which has been supplied by the author(s)

Supplement 2: Acute and follow-up CMR of post-COVID-19 athletesshowing alterations on CMR scan

Athlete No.	Sex	*Symptoms*	CMR findings	CMR images	Time to follow-up CMR from COVID-19 (days)	Follow-up CMR findings	Follow-up CMR images
1.	Male	Moderate<br>• headache<br>• fever<br>• chest pain<br>• joint pain<br>• diarrhea<br>• smell and taste disturbance	LVEF: 52 %<br>GLS: -18 %<br>Septal native T1: normal<br>Septal native T2: normal<br>Pathological LGE / pattern: Yes - Lateral subepicardial LGE (5SD method): 9.3%	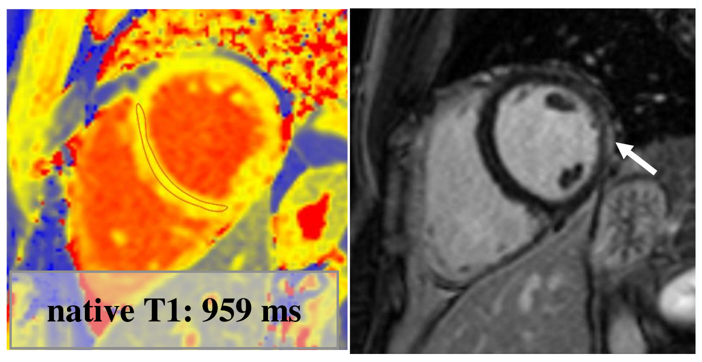	299	LVEF: 58 %<br>GLS: -20 %<br>Septal native T1: normal<br>Septal native T2: normal<br>Pathological LGE / pattern: Yes - Lateral subepicardial LGE (5SD method): 5.4%	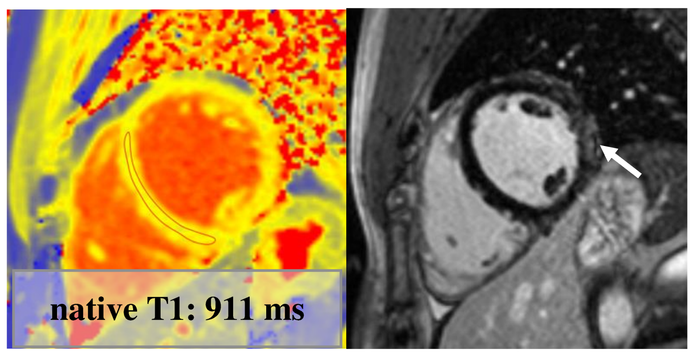
4.	Female	Long- COVID<br>• palpitation<br>• long lasting fatigue	LVEF: 67 %<br>GLS: - 27 %<br>Septal native T1: gray zone normal/elevated<br>Septal native T2: elevated<br>Pathological LGE / pattern: No	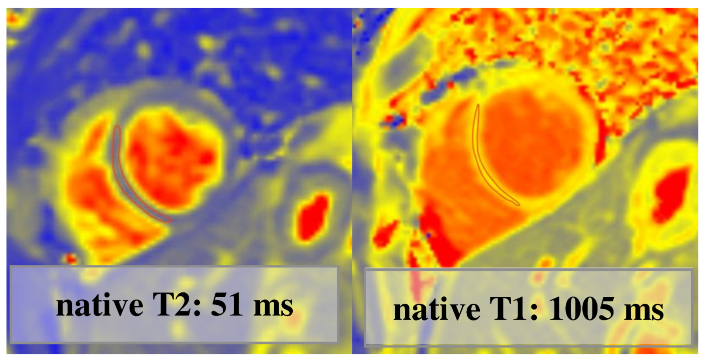	129	LVEF: 62 %<br>GLS: - 27 %<br>Septal native T1: normal<br>Septal native T2: normal<br>Pathological LGE / pattern: No	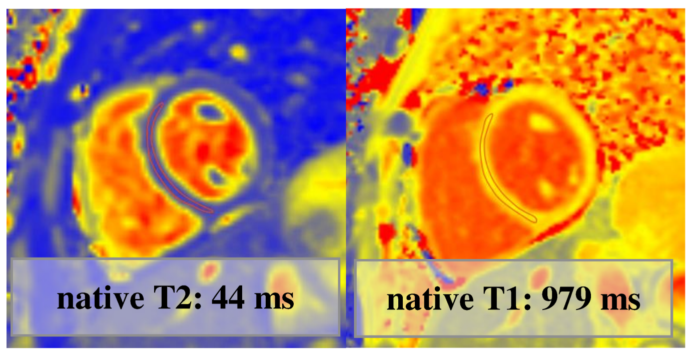
5.	Female	Moderate<br>• chest pain<br>• back pain<br>• smell and taste disturbance	LVEF: 60 %<br>GLS: -22 %<br>Septal native T1: elevated<br>Septal native T2: elevated<br>Pathological LGE / pattern: No	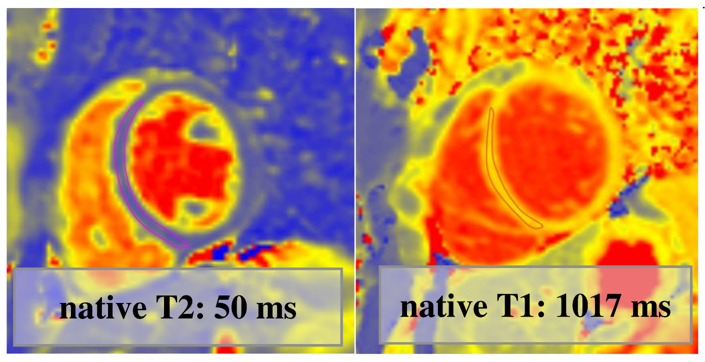	105	LVEF: 62 %<br>GLS: -25 %<br>Septal native T1: normal<br>Septal native T2: normal<br>Pathological LGE / pattern: No	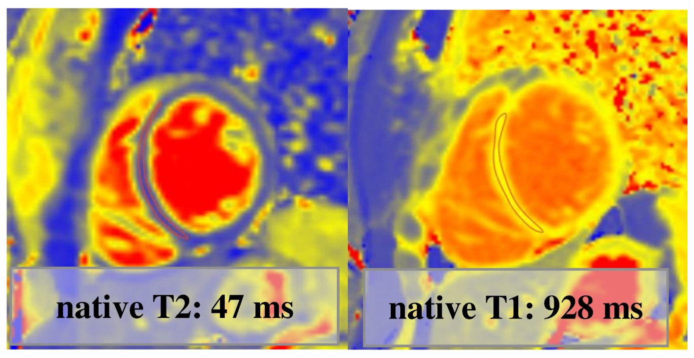
6.	Female	Mild<br>• fever<br>• fatigue<br>• palpitation<br>• smell and taste disturbance	LVEF: 55 %<br>GLS: -18 %<br>Septal native T1: elevated<br>Septal native T2: normal<br>Pathological LGE / pattern: No	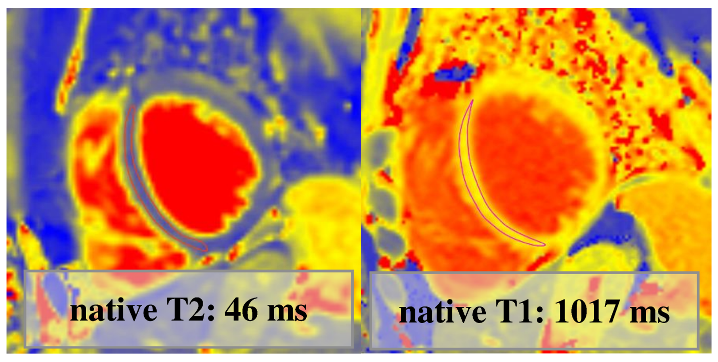	81	LVEF: 57 %<br>GLS: -21 %<br>Septal native T1: gray zone normal/elevated<br>Septal native T2: normal	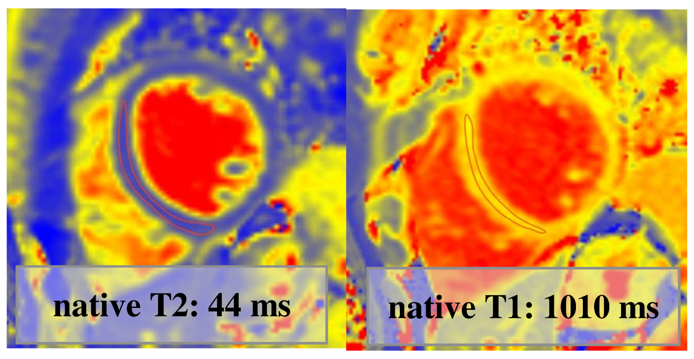
